# Potential Role of CXCR4 Targeting in the Context of Radiotherapy and Immunotherapy of Cancer

**DOI:** 10.3389/fimmu.2018.03018

**Published:** 2018-12-21

**Authors:** Franziska Eckert, Karin Schilbach, Lukas Klumpp, Lilia Bardoscia, Efe Cumhur Sezgin, Matthias Schwab, Daniel Zips, Stephan M. Huber

**Affiliations:** ^1^Department of Radiation Oncology, University Hospital Tuebingen, Tuebingen, Germany; ^2^Department of General Pediatrics/Pediatric Oncology, University Hospital Tuebingen, Tuebingen, Germany; ^3^Dr. Margarete Fischer-Bosch-Institute of Clinical Pharmacology, Stuttgart, Germany; ^4^Department of Radiation Oncology, University of Brescia, Brescia, Italy; ^5^Departments of Clinical Pharmacology, Pharmacy and Biochemistry, University Hospital and University Tuebingen, Tuebingen, Germany

**Keywords:** immunotherapy, cancer radiotherapy, CXCR4, SDF-1 (CXCL12), T cells, dendritic cells, NK cells, regulatory T cells

## Abstract

Cancer immunotherapy has been established as standard of care in different tumor entities. After the first reports on synergistic effects with radiotherapy and the induction of abscopal effects—tumor shrinkage outside the irradiated volume attributed to immunological effects of radiotherapy—several treatment combinations have been evaluated. Different immunotherapy strategies (e.g., immune checkpoint inhibition, vaccination, cytokine based therapies) have been combined with local tumor irradiation in preclinical models. Clinical trials are ongoing in different cancer entities with a broad range of immunotherapeutics and radiation schedules. SDF-1 (CXCL12)/CXCR4 signaling has been described to play a major role in tumor biology, especially in hypoxia adaptation, metastasis and migration. Local tumor irradiation is a known inducer of SDF-1 expression and release. CXCR4 also plays a major role in immunological processes. CXCR4 antagonists have been approved for the use of hematopoietic stem cell mobilization from the bone marrow. In addition, several groups reported an influence of the SDF-1/CXCR4 axis on intratumoral immune cell subsets and anti-tumor immune response. The aim of this review is to merge the knowledge on the role of SDF-1/CXCR4 in tumor biology, radiotherapy and immunotherapy of cancer and in combinatorial approaches.

## Introduction

In radiation oncology, chemokine receptor CXCR4 and its ligand SDF-1 (stromal cell derived factor-1, CXCL12) have been described as prognostic factor for head and neck squamous cell carcinoma [e.g., (1)]. Functional data in glioblastoma models point to a role in migration and invasion of cancer cells (2). These and other observations strongly suggest SDF-1/CXCR4 signaling as promising target in anti-cancer therapy, in particular, in combination with radiation therapy (3). However, clinical development of CXCR4 antagonists has mainly focussed on mobilization of hematopoietic stem cells from the bone marrow to peripheral blood (4).

Radiation therapy has proven to elicit both pro-inflammatory, immunostimulatory activities, and anti-inflammatory, immunosuppressive mechanisms. These effects are dependent on radiation dose, tumor biology and the host predisposition (5). As immunotherapy for cancer has been established as standard of care for several cancer entities, such as melanoma (6) and lung cancer (7), the immunologic effects of standard anti-cancer treatment, such as radiation therapy and targeted therapies are of major interest. Radiation-induced immune modulation has been described as direct effects on irradiated tumor cells (“on-target” immunogenic effects) as well as indirect effects in the tumor immune microenvironment (“off-target” effects) (8). Remarkably, recent data also link CXCR4 blockade with antitumor immunity in the tumor immune microenvironment suggesting SDF-1/CXCR4-targeting as a therapeutic tool to interfere with the immune system.

The present article, therefore, aims to give an overview about the plethora of functions of SDF-1/CXCR4 signaling in tumor biology and immune responses in the context of combined radiotherapy and immunotherapy. The knowledge about these functions is indispensable for developing new concepts of anti-cancer therapy that comprise radiotherapy, immunomodulation and SDF-1/CXCR4 targeting.

## Interference of Ionizing Radiation With Immune Responses

Radiotherapy effects on cancer had been mostly attributed to direct cytotoxic effects on cancer cells (especially DNA damage) (9). With the advance of cancer immunotherapies preclinical and clinical observations pointed toward synergistic effects. The so-called “abscopal effect” describes responses to radiotherapy (mostly in combination with immunotherapy) outside the irradiated volume and has been linked to immune mechanisms (10). The combination of immune checkpoint inhibitors with local tumor irradiation seems to induce synergistic effects and is currently tested in multiple clinical trials (11, 12). In addition, theoretical rationales and preclinical data provide the basis for also combining radiotherapy with other immunotherapy strategies, such as anticancer-vaccines and cytokine-based therapies (13).

### Immune Effects of Tumor Irradiation

The mechanisms of radiation-induced immune effects have been summarized as immune-stimulating and immunosuppressive either directly in tumor cells or in the microenvironment (8). Radiation triggers anti-tumor immune responses directly in the cancer cells by upregulation of MHC-I molecules (14, 15) and possible induction of new tumor associated antigens (14). Cell death mechanisms induced by tumor irradiation lead to “immunogenic cell death” (ICD) (16, 17) characterized by the ability to stimulate the innate immune system and thus indirectly also adaptive immune responses (18, 19). ICD is characterized by the release of danger associated molecular patterns (DAMPs), such as calreticulin (20), high-mobility-group-box 1 (HMGB1) (21) and extracellular adenosine-tri-phosphate (ATP) (22). Additional mechanisms include cytokine release, such as CXCL10 (23), and type-1 interferon (24). Indirect immune stimulation has been attributed to increase and activation of tumor-infiltrating lymphocytes (25, 26), as well as maturation of dendritic cells (DCs) (27). The fact that clinically relevant anti-tumor responses (e.g., abscopal effects after palliative radiotherapy in metastatic cancer patients) are rare despite of these strong immune-stimulating effects is most probably due to simultaneously induced immunosuppression by tumor irradiation. Irradiated tumor cells upregulate immune checkpoint molecules, such as PD-L1 (28, 29) and release immunosuppressive cytokines, such as TGFβ (30, 31). Immunosuppressive cells increased in the tumor immune microenvironment upon local irradiation include regulatory T cells (32, 33) and myeloid-derived suppressor cells (MDSC) (34–36).

### Combination Therapies of Irradiation and Immunotherapy for Cancer

These mechanisms have become the basis for combining radiotherapy and immunotherapy in order to exploit pro-immunogenic properties of irradiation and block immunosuppressive actions. Clinical trials ongoing with combinatorial approaches include immune checkpoint inhibition, cytokine based therapies and vaccination strategies (37, 38). Combination of radiotherapy with immune checkpoint inhibition has been recently summarized (39). Besides its use in metastatic cancer patients, durvalumab as adjuvant treatment after definitive radiochemotherapy for non-small cell lung cancer has shown significantly improved disease free survival (40). Vaccination strategies used in combination with radiotherapy include peptide and mRNA based approaches (41–43) which showed promising results in syngeneic mouse xenograft models. IL2 and IL12 as tumor targeted immunocytokines have been tested in combination with tumor irradiation in *in vivo* models showing promising results (44–47).

In conclusion, the strong rationale and promising results led to an increasing use of immunotherapeutics in combination with local tumor irradiation in standard of care treatment of palliative cancer patients as well as in numerous clinical trials with high expectations of the oncological field to improve survival and prognosis of cancer patients.

## SDF-1/CXCR4 Function In Tumor Biology

SDF-1/CXCR4 signaling has been shown to contribute to virtually all processes in tumor biology. As described in this section, SDF-1/CXCR4 signaling reportedly contributes to neoplastic transformation, malignant tumor progression, infiltration, metastasis, angiogenesis and vasculogenesis, and consequently therapy resistance of many different tumor entities.

### CXCR4, a Marker of Cancer Stem(-Like) Cells or Tumor-Initiating Cells

CXCR4 chemokine receptors are expressed by hematopoietic stem cells and are required for the trapping of these cells within the stem cell niches of the bone marrow. CXCR4 antagonists, such as AMD3100 (Plerixafor), therefore, can be used to mobilize stem cells into the peripheral blood for hematopoietic stem cell donation (see below). Beyond that, SDF-1/CXCR4 signaling has been shown to be functional in neural progenitor cells and to direct neural cell migration during embryogenesis (48). Notably, CXCR4 expression is further upregulated when neural progenitor cells differentiate into neuronal precursors whereas SDF-1 is upregulated during maturation of neural progenitor cells into astrocytes. While CXCR4 is localized in the cell body of neuronal precursors, expression is primarily restricted to axons and dendrites in mature neurons (49). In addition, SDF-1/CXCR4 signaling has been reported to contribute to chemotaxis and differentiation into oligodendrocytes of engrafted neural stem cells resulting in axonal remyelination in a mouse model of multiple sclerosis (50). Together this suggests that neurogenesis requires functional SDF-1/CXCR4 signaling and CXCR4 as marker of especially the neuronal lineage of neural stem cells.

Primary glioblastoma multiforme (GBM) develops directly by neoplastic transformation of neural stem cells and not by malignant progression from astrocytic gliomas or oligodendroglomas (the latter two are characterized by mutations in the IDH genes). Not unexpectedly, stem(-like) subpopulations of GBM functionally express SDF-1/CXCR4 signaling (51–56). Notably, auto-/paracrine SDF-1/CXCR4 signaling is required for maintenance of stemness and self-renewal capacity (57–59) since SDF-1/CXCR4 targeting leads to loss of stem cell markers and differentiation of stem(-like) cells into “differentiated” tumor bulk.

Besides glioblastoma, SDF-1/CXCR4 signaling has been shown to be functional in stem(-like) subpopulations of retinoblastoma (60), melanoma (61), pancreatic ductal adenocarcinoma (62), non-small cell lung cancer (63), cervical carcinoma (64), prostate cancer (65), head and neck squamous cell carcinoma (66), rhabdomyosarcoma (67, 68), synovial sarcoma (56), and leukemia (69). In summary, these data might hint to an ontogenetically early onset of SDF-1/CXCR4 signaling in mesenchymal and epithelial primordia of the different organs which might be the reason for SDF-1/CXCR4 expression in stem(-like) subpopulations of many different tumor entities.

Transition of stem(-like) cells and differentiated tumor bulk and *vice versa* seems to be highly dynamic and regulated by the reciprocal crosstalk with untransformed stroma cells of the tumor microenvironment (70–72). Beyond that, this crosstalk seems to induce phenotypical changes of cancer stem(-like) cells as deduced from the following observation. Sorted CD133^+^ stem(-like) cells and CD133^−^ differentiated bulk cells of GBM did not differ in repair of radiation-induced DNA double strand breaks *in vitro*. Upon orthotopic transplantation in immunocompromized mice, however, CD133^+^ cells repaired more efficiently than CD133^−^ cells indicating tumor-microenvironment-mediated upregulation of DNA repair selectively in CD133^+^ GBM cells (73). The next paragraph introduces the impact of SDF-1/CXCR4 signaling on the crosstalk of tumor cells with non-transformed stroma cells and its function for the cancer stem(-like) cell phenotype.

### SDF-1/CXCR4 Signaling in the Crosstalk of Cancer Stem(-Like) Cells With Non-transformed Stroma Cells

The functional significance of SDF-1/CXR4 signaling between tumor cells and the tumor stroma is suggested by a retrospective analysis of genetic SDF-1 variants in patients with colorectal cancer where a certain SDF-1 polymorphism in fibroblasts is associated with higher stromal SDF-1 expression and increased risk for lymph node metastases in stage T3 colorectal cancer (74). Moreover, diffuse-type gastric cancer probably develops from quiescent Mist1^+^ stem cells upon Kras and APC mutation and loss of E-cadherin. Most importantly, this seems to be dependent on inflammatory processes triggered by SDF-1-expressing endothelial cells and CXR4-expressing gastric innate lymphoid cells that form the perivascular gastric stem cell niche (75).

Likewise, GBM cells co-opt vessels and home to perivascular stem cell niches. Reciprocal signaling between endothelial and GBM cells within these niches has been shown to induce and maintain a stem(-like) cell phenotype of GBM cells on the one hand and to promote angiogenesis on the other [for review see (76)]. Moreover, trans-differentiation of GBM stem-like cells into endothelial cells (77, 78) and pericytes (79) contributes to the adaptation of the tumor microvasculature to the needs of the GBM cells. SDF-1/CXCR4 signaling reportedly exerts pivotal functions in these processes. In particular, CXCR4 on GBM cells and SDF-1 produced by endothelial cells direct perivascular invasion as demonstrated *in vitro* and in orthotopic glioma mouse models (79–81). Accordingly, SDF-1-degradation by the cysteine protease cathepsin K facilitates evasion of GBM cells out of the niches (82). In addition to chemotaxis, CXCR4 stimulation by SDF-1 induces the production of vascular endothelial growth factor (VEGF) in GBM (83) and especially in CD133^+^ GBM stem-like cells (84). VEGF, in turn, stimulates beyond angiogenesis upregulation of CXCR4 (85) and SDF-1 (86) in microvascular endothelial cells. Moreover, VEGF is required for trans-differentiation of GBM-derived progenitor cells into endothelial cells (77). The significance of targeting VEGF and SDF-1/CXCR4 signaling for stem cell niche formation can be deduced from the observation that targeting of both, VEGF and CXCR4, decreases the number of perivascular GBM cells expressing stem cell markers in an orthotopic glioma mouse model, which was associated with improved survival of the tumor-bearing mice (87).

A further example of up-regulation of a stem(-like) cell phenotype mediated by SDF-1 signaling was reported for breast cancer cells where SDF-1 release from tumor-associated fibroblasts is required for the maintenance of tumor initiation capability (88). Finally, leukemia cells have been demonstrated to be trapped in stem cell niches of the bone marrow (89–91), and follicular lymphoma stem(-like) cells to follicular DCs in the germinal center of lymph nodes (92) by SDF-1/CXCR4 signaling. Combined, these data suggest that SDF-1 directed chemotaxis to certain microenvironmental stem cell niches is a general phenomenon of CXCR4-expressing hematopoietic and non-hematopoietic cancer cells. Of utmost importance, interactions with stromal cells within these niches contribute to a malignant and therapy-resistant phenotype of niche-residing cancer cells as outlined in the next paragraph.

### SDF-1/CXCR4 Signaling in Tumor Microenvironment-Induced Therapy Resistance of Cancer Stem(-Like) Cells

Subventricular zones (SVZs) of the brain accommodate neural stem cells and have been shown to attract human GBM stem(-like) cells through SDF-1/CXCR4 signaling in an orthotopic glioma mouse model (93). Importantly, SVZ residence induces radioresistance of GBM stem(-like) cells in direct dependence on SDF-1 release by the SVZ stromal cells (94). Evidence for radioresistance conferred by SDF-1/CXCR4-dependent residency in perivascular niches was further provided by the observation that CXCR4 knockdown in mouse GBM cells resulted in both, diminished perivascular invasion and increased radiosensitivity (81).

Likewise, in mouse models of acute myeloid leukemia CXCR4 antagonism mobilized leukemia cells out of the bone marrow niches and, at the same time, enhanced chemosensitivity (90, 91). Mechanistically, bone marrow mesenchymal cells have been demonstrated to upregulate a signaling complex in the leukemia cells comprising CXCR4 and activating pro-survival signals via extracellular signal-related kinase 1/2 (ERK1/2) and the phosphoinositide 3-kinase (PI3K)/Akt pathway (95). Moreover, bone marrow disseminated *xeno*grafted head and neck squamous cell carcinoma (HNSCC) exhibits a higher cisplatin resistance than lung metastases *ex vivo*. This difference critically depends on TGF-β-triggered SDF-1/CXCR4 signaling (96). In summary, these preclinical *in vivo* and *ex vivo* data strongly suggest that SDF-1/CXCR4-mediated residency of tumor cells in stem cell niches induces resistance to chemo- and/or radiation therapy probably by inducing expression of a therapy-resistant cancer stem(-like) cell phenotype. The maintenance of the latter—as discussed above and impressively demonstrated by the *ex vivo* comparison of bone marrow and lung disseminated HNSCC—itself crucially depends on SDF-1/CXCR4. Beyond cancer stem(-like) cell induction, SDF-1/CXCR4 signaling has been demonstrated to trigger tumor invasion and metastasis as discussed in the next chapter.

### SDF-1/CXCR4 Signaling in Triggering Tumor Invasion and Distant Metastasis

Associations between SDF-1/CXCR4 polymorphisms or SDF-1/CXCR4 abundance in tumor specimens and clinical outcome in several but not all studies might suggest a role of SDF-1/CXCR4 signaling in metastatic progression in a variety of tumor entities, such as renal cell carcinoma (97), prostate cancer (98), HNSCC (99–102), esophagogastric cancer (103), colorectal cancer (74), hepatocellular carcinoma (104), or osteosarcoma (105). In preclinical studies, overexpression of CXCR4 has been demonstrated to dramatically increase lung and liver metastases of murine pancreatic cancer in tail vein metastasis assays in nude mice (106). Consistently, antagonizing CXCR4 inhibited lung metastasis of human tongue squamous cell carcinoma (107), esophageal cancer (108), breast cancer (109) in immunocompromized mice. Intra-arterially injected circulating CXCR4-expressing melanoma cells require SDF-1 signaling by mesenchymal stem cells that act as pericytes for extravasation to bone and liver and perivascular niche formation as demonstrated by humanized heterotopic bone formation assay (110). Combined, these examples suggest that CXCR4 expression by cancer cells contribute to their tropism to metastatic niches.

Along those lines, CXCR4 downregulation by overexpression of miR-613 reportedly inhibits lung metastasis of osteosarcoma orthotopically *xeno*grafted in nude mice (105). Notably, epigenetic downregulation of SDF-1 has been demonstrated to boost metastases of CXCR4-expressing sarcoma in mouse models (111). Likewise, a SDF-1 polymorphism with low SDF-1 expression in breast cancer has been proposed to associate with susceptibility to metastases (112). It is, therefore, tempting to speculate that SDF-1^−^/CXCR4^+^ tumor cells are particularly prone to metastasize. As a matter of fact, high CXCR4 and low SDF-1 expression by the tumor has been associated with poor overall survival in osteosarcoma (111) and metastasis-free survival in head and neck squamous cell carcinoma (113). The latter association, however, was not confirmed by a recent study (114). Nevertheless, these reports strongly suggest a pro-metastatic function of SDF-1/CXCR4 signaling in several cancer entities.

In GBM which usually does not metastasize outside the central nervous system, SDF-1/CXCR4 signaling has been demonstrated *in vitro* to exert pivotal function in cell migration and chemotaxis (115–117). Most probably, SDF-1/CXCR4-dependent migration/chemotaxis does not only contribute to the above discussed homing of GBM cells to protective perivascular stem cell niches (see above) but also to deep infiltration of the brain parenchyma by highly migratory GBM stem(-like) cells. One driver of glioblastoma dissemination might be hypoxia through HIF-1α mediated up-regulation of SDF-1 and CXCR4 in GBM cells (85, 86). Unexpectedly, VEGF- or VEGF-R-targeting has been demonstrated *in vitro* to directly up-regulate CXCR4 expression and chemotaxis toward SDF-1 in VEGF-R-expressing GBM cells in a TGFβ-dependent manner (118). In accordance with these observations, anti-angiogenic therapy of orthotopic mouse glioma promotes GBM invasion by CXCR4 upregulation. Additional CXCR4-targeting blunts this effect (119). Consistently, combined VEGF- or VEGF-R- and CXCR4 antagonism prolongs survival of mice bearing orthotopically *xeno*grafted GBM as compared to only VEGF/VEGF-R-targeted mice (87, 118, 120). Also along those lines, anti-angiogenic therapy, such as Bevacizumab which has been demonstrated in large clinical trials not to improve overall survival of GBM patients is under suspicion to foster distant spread of the tumor at recurrence (121). Even if the tumor spread-promoting effect of Bevacizumab is under debate (122), nevertheless, these data bear witness to a close interaction between tumor hypoxia and SDF-1/CXCR4 signaling as introduced in more detail in the next chapter.

### SDF-1/CXCR4-Signaling and its Function for Vasculogenesis

Hypoxia-induced up-regulation of SDF-1 secretion in tumors reportedly stimulates homing and engraftment of bone marrow-derived myeloid cells, as well as mesenchymal stem cell-derived endothelial and pericyte progenitor cells. This recruitment promotes neovascularization of the hypoxic tumor by transition of the myeloid and progenitor cells into endothelium and pericytes. Such SDF-1/CXCR4-dependent vasculogenesis has been demonstrated in mouse models of several tumor entities, such as GBM (123–127), HNSCC (128), lung adenocarcinoma (129), hepatocellular carcinoma (130) or breast cancer (131). Importantly, irradiation has been shown to induce SDF-1 expression and thus may boost vasculogenesis and tumor re-growth after end of therapy (125, 131–133) suggesting a radioresistance-conferring action of SDF-1/CXCR4 signaling as discussed in the next paragraph.

### SDF-1/CXCR4-Signaling and Radioresistance

In many tumor entities radiation therapy is part of standard of care. Ionizing radiation has been demonstrated *in vitro* as well as *in vivo* to stimulate SDF-1/CXCR4 signaling in different human and mouse tumor entities either directly by S-nitrosylation and stabilization of HIF-1α (134) or indirectly via radiation-induced endothelial cell killing and resulting hypoxia (135) or HIF-1α-independent mechanisms (136). Radiation-induced modifications in SDF-1/CXCR4 signaling, in turn, have been reported in gliomas (116, 125, 127, 137), mesotheliomas (138), prostate (139), cervical (140), lung (131, 141) and breast cancer (131). Aside from the direct effect on cancer cells, radiation-induced SDF-1 secretion is also observed in different normal tissues/cells (94, 136, 142–147) or cancer-associated fibroblasts (144).

Importantly, radiation-modulated SDF-1/CXCR4 signaling has been shown to stimulate tumor re-growth (142, 148), EMT (144), migration (116), invasiveness (81, 127, 138, 141, 144) and metastases (138, 145), as well as homing of hematopoietic progenitor cells and accelerated vasculogenesis (125, 127, 131–133, 136, 137, 142). Thus, radiation-induced SDF-1/CXCR4 signaling may foster radioresistance, malignant progression and recurrence of tumors (94, 125, 139, 149–151). Preclinical evidence shows reduced metastases in orthotopic murine models of cervical cancer with Cisplatin-based radiochemotherapy and AMD3100 (152).

Thus, as CXCR4 is a prognostic marker for local control after curative radiotherapy and irradiation interferes with SDF-1/CXCR4 signaling, there is a strong rationale to develop translational and clinical interventional studies combining CXCR4 targeting with curative radio(chemo)therapy. The roles of SDF-1/CXCR4 signaling in tumor biology are summarized in Table 1.

**Table 1:** (Patho)physiological role of SDF1/CXCR4 signaling and targeting in cancer.

**Cell type**	**(Patho)physiological role of SDF-1/CXCR4 signaling**	**Effects of CXCR4 targeting**	**References**
Cancer cells	VEGF production in GBM		(83)
		Mobilization of leukemia cells from BM, enhanced chemosensitivity	(90, 91)
	Association with decreased patient survival	Decreased metastasis formation	(97–105, 111)
	Cell migration in GBM		(115–117)
		Inhibition of VEGF-mediated migration in GBM, prolonged survival of mice	(119)
	Vasculogenesis		(123–133)
	Radiation-induced EMT		(143)
	Radiation-induced invasiveness		(81, 127, 138, 141, 144)
Cancer stem(like) cells (CSC)	Maintenance of stemness, self renewal capacity		(51–56)
		Loss of stem cell markers, differentiation to “bulk” cells	(57–69)
	VEGF production in GBM CSCs		(84)
	Attraction to subventricular stem cell niches		(93, 94)
Stroma cell/cancer cell crosstalk	SDF-1 in fibroblasts increases lymph node metastases in CRC		(74)
	SDF-1 in endothelial cells contributes to gastric cancer development		(75)
	Perivascular invasion of GBM	Reduction of perivascular GBM cells, increased radiosensitivity	(79–81, 87)
	SDF-1 in fibroblasts required for tumor initiation in BC		(88)

*BC, breast cancer; BM, bone marrow; CRC, colorectal cancer; CSC, cancer stem(like) cell; EMT, epithelial mesenchymal transition; GBM, glioblastoma multiforme; VEGF, vascular endothelial growth factor*.

### SDF-1/CXCR4 Signaling as Druggable Target in Anti-cancer Therapy

As already touched upon, retrospective clinical data might hint to associations between SDF-1 polymorphisms or SDF-1/CXCR4 expression levels with susceptibility to neoplastic transformation, malignant progression or therapy response in a variety of tumor entities, such as renal cell carcinoma (97), prostate cancer (98), HNSCC (1, 100, 102, 113, 114), esophagogastric cancer (103), hepatocellular carcinoma (104), colorectal cancer (74), breast cancer (153), osteosarcoma (111), low grade glioma (154, 155), or GBM (156, 157). Beyond cancer SDF-1 genetics has been associated with e.g., the pathogenesis of multiple sclerosis (158) or prognosis in patients with cardiovascular disease (159).

**Figure 1:**
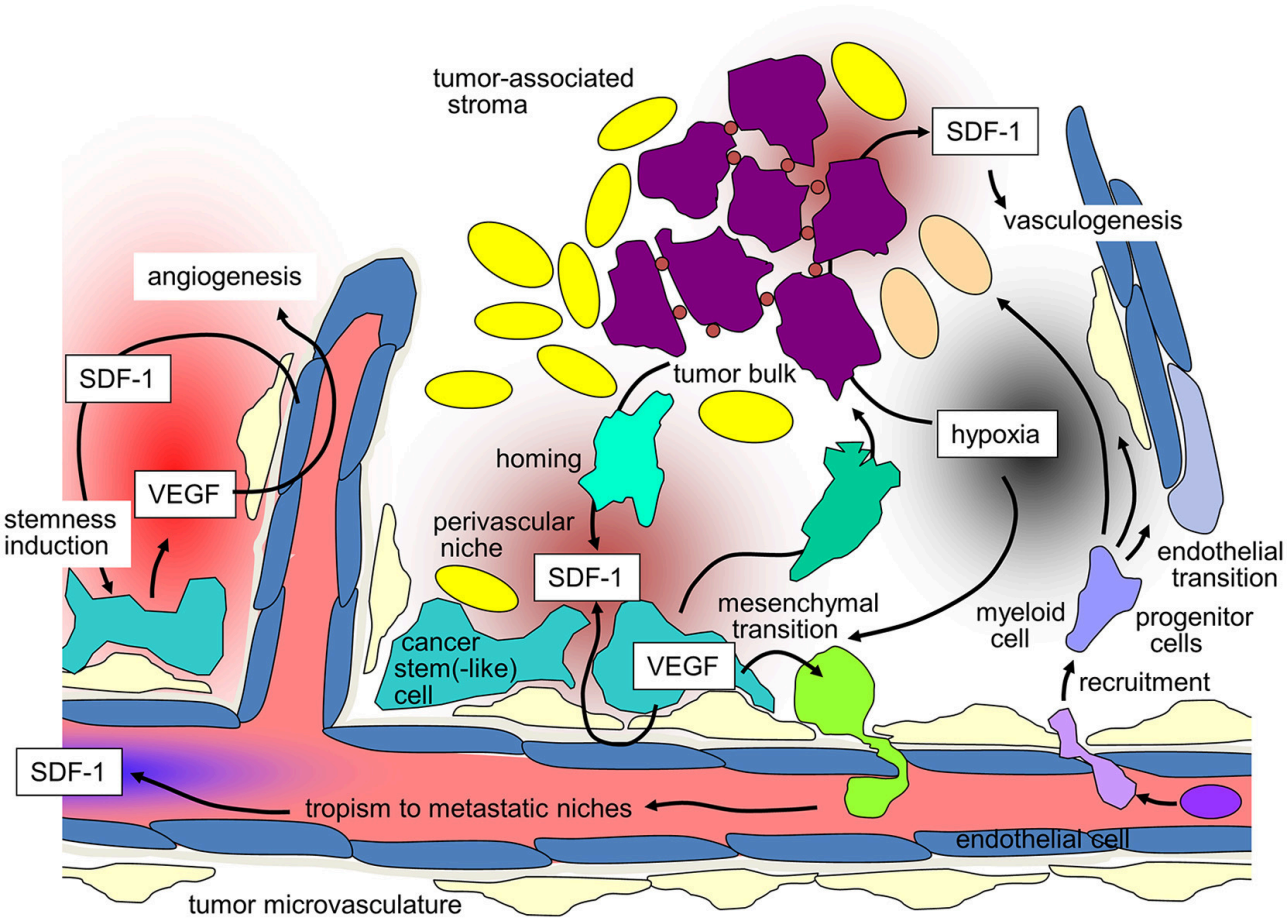
SDF-1/CXCR-4 signaling in tumors and its contribution to maintenance of tumor stemness, recruiting of distant stroma cells, angio- and vasculogenesis, and metastasis (for details see text).

Apart from genetic variants, SDF-1 as well as CXCR4 were shown to be regulated epigenetically by DNA methylation. DNA methylation status of the genes was suggested as prognostic biomarkers for e.g., breast or pancreatic cancer and GBM (160–162). Such prognostic or predictive value of SDF-1/CXCR4 might be expected from the plethora of SDF-1/CXCR4 functions in tumor biology mentioned above. These functions contribute to malignancy, progression and therapy resistance of the tumors and render SDF-1/CXCR4 signal to an ideal target in anti-cancer therapy. In particular, a combination of SDF-1/CXCR4-targeting and radiotherapy seems to be promising given the above mentioned radioprotective functions of SDF-1/CXCR4 signaling (Figure 1). Moreover, combinatorial treatment of conventional chemotherapy with CXCR4 inhibitors might be an approach to overcome cancer therapy resistance (163).

In fact, several SDF-1- or CXCR4-targeting drugs have been applied in preclinical models [e.g., Ulocuplumab (164), ALT-1188 (165), POL5551 (166), PRX177561 (167)], were well-tolerated in clinical trials [e.g., AMD070 (168), Balixafortide (POL6326, Polyphor) (169)] or are FDA-approved [AMD3100, Plerixafor (170)] indicating that SDF-1/CXCR4 targeting is clinically feasible. Overall, Plerixafor used for stem cell mobilization does not induce severe side effects (171, 172). A randomized phase 3 trial comparing G-CSF with plerixafor vs. placebo reported mainly fatigue, gastrointestinal side effects like nausea and diarrhea and injection site reactions (173).

CXCR4, however, is expressed on immune cells suggesting that SDF-1/CXCR4-targeted anti-cancer therapy at the same time interferes with the immune response to cancers and cancer cells in e.g., circulation or micrometastases. In order to explore these functions and develop a rationale if trimodal therapy combining CXCR4 targeting with immunotherapy and radiotherapy might be of benefit, it is crucial to understand the function of SDF-1/CXCR4 signaling in immune cells and the effects of CXCR4 inhibition on the immune response to cancer.

## Physiologic Role of Cxcr4 in the Immune System

In addition to its function in tumor biology, SDF-1/CXCR4 signaling controls multiple physiological processes in hematopoiesis, T, B and NK cell development and the organization of the immune system. Ablation of either of the components of the SDF-1/CXCR4 axis generates a similar phenotype of deficient B lymphopoiesis and myelopoiesis, disturbed immune responses leading to cancers, autoimmunity and inflammatory diseases (174–176). Recently a 16 amino acid fragment of serum albumin (EPI-X4) was identified as an effective and highly specific endogenous CXCR4 antagonist (177). Peptide EPI-X4 is evolutionarily conserved and generated from the highly abundant albumin precursor by pH-regulated proteases. It antagonizes SDF-1-induced tumor cell migration and suppresses inflammatory responses in mice. In human the peptide is abundant in the urine of patients with inflammatory diseases. EPI-X4 mobilizes stem cells, which explains in part why stem cells can directly respond to inflammation with their migration into the periphery.

### Hematopoietic Stem Cell Niche

HSCs (Hematopoietic stem cells) are a rare cell population that can give rise to all lineages of the immune system. HSCs reside in the undifferentiated state in the bone marrow, where the binding of their CXCR4 receptor to its ligand SDF-1—constitutively provided by the bone marrow (BM) niche—promotes their survival (178, 179) while negatively regulating their proliferation (180–182). In addition to direct effects on HSCs, SDF-1/CXCR4 signaling also promotes survival and growth of human bone marrow stromal stem cells (183). Inhibiting the interaction between CXCR4 receptor and SDF-1 leads to the migration of hematopoietic stem and progenitor cells (HSPC) into the periphery, a process termed mobilization, which is required for harvesting stem cells for transplantation [either autologous from the patient (184) or in healthy donors (185)]. A dramatic increase in mobilization efficiency and yields of progenitor cells compared to standard G-CSF is achieved when using CXCR4 antagonists, such as AMD3100, Mozobil® (184, 186, 187). CXCR4 antagonist BL-8040 in a recent phase I clinical study (NCT02073019) besides highly efficient mobilization of pluripotent hematopoietic progenitors showed also superior yields of CD4^+^ and CD8^+^ T cells, NKT, NK, and DCs, suggesting increased engraftment ability of CXCR4 mobilized populations, a higher anti-tumor effect and faster immune reconstitution potential. Moreover, mobilization as a 1-day procedure is less wearing for the donor and allows faster access to the stem cells (188). Since HSCs maintain hematopoiesis throughout life, qualitative and quantitative effects through prolonged pharmacologic blockade of the SDF-1/CXCR4 axis need to be investigated. Concerns that the HSC pool in the bone marrow would decrease were not confirmed, as CXCR4-blockade led to higher repopulating capacity and exceptional mobilization along with an expansion of the BM HSC pool, which unexpectedly suggests reversible inhibition of the SDF-1/CXCR4 axis also as a novel strategy to restore damaged BM (189). BM HSCs during reversible long-term CXCR4/SDF1 long term blockade increase their cycling activity 2- to 3-fold [only 10–20% of Lin-Sca1+Kit- (LSK) and 30–40% of LSK SLAM cells being quiescent (G0 phase)] compared to 50–60% of LSK and 70% of LSK SLAM under homeostatic conditions (190, 191). Thus, these findings together with mounting evidence for direct cytolytic and specific anti-leukemic effects of CXCR4 inhibition (192–194) suggests prolonged CXCR4 blockade as a novel safe therapeutic scheme for treatment of (hematologic) malignancies either alone or in conjunction with chemotherapy.

### Dendritic Cells

The priming of naïve T cells is dependent on efficient antigen presentation and a strong costimulatory signal both provided by dendritic cells during Th1 polarized immune responses. Th1 polarization is thought to be critical for immune rejection of tumors, while activated T cells polarized to Th2 cytokine and cell profiles might induce even tumor immune evasion (195). Plasmacytoid DCs (pDCs) as type I interferon (IFN)-producing cells play a central role in antiviral and anti-tumor immunity. pDCs are produced from hematopoietic stem cells in the bone marrow where they nestle down with reticular cells in the intersinal space which abundantly provides SDF-1. Concordantly, the number of pDCs and their earliest progenitors is severely reduced in the absence of CXCR4 *in vitro* and *in vivo*, underlining the function of SDF-1/CXCR4 axis as a key regulator of pDC development and the importance of provision of SDF-1 by cellular niches (196, 197). Upon activation, CXCR4 expressing DCs migrate into SDF-1 expressing lymphatic vessels where they initiate immune responses, a process that is severely blocked by systemic CXCR4 antagonist application (198). Since the dysregulated expression of SDF-1/CXCR4 is associated with the pathology of various autoimmune diseases, including rheumatoid arthritis, systemic lupus erythematosus, and multiple sclerosis, targeting SDF-1/CXCR4 axis with 4-F-Benzoyl-TN14003 may be beneficial for prevention of autoimmune disease (198–201). It is not clear, whether these effects on DCs might decrease the efficacy of anti-cancer immune responses upon CXCR4 inhibition.

### Myeloid Derived Suppressor Cells

As reviewed in (202), MDSCs are highly immunosuppressive cells in the tumor microenvironment and mainly suppress intratumoral T cells. SDF-1 secreted by tumor associated fibroblasts induces MDSCs and impairs anti-tumor immune responses as shown in a hepatic carcinoma model (203). Another liver cancer model (metastases of colorectal carcinoma) showed less MDSC infiltrates in the metastases after treatment with AMD3100, accompanied by reduced metastases growth (204). Patient samples of ascites also showed that CXCR4 signaling is involved in MDSC recruitment. SDF-1 release of cancer cells as well as CXCR4 signaling in MDSCs could be abrogated by COX2 inhibition.

### Regulatory T Cells (nTreg and iTreg)

Regulatory T cells (Tregs) constitute 5–10% of peripheral CD4^+^ T cells in humans (205, 206). Tregs maintain immune homeostasis, peripheral tolerance and prevent autoimmunity by suppressing and terminating immune responses. They constitute a major barrier for an effective antitumor immunity, and the number of peripheral and intratumoral Treg cells is an independent prognostic factor in malignancies (207). Cancer cell- and M2 macrophages derived SDF-1 attracts Treg cells into the tumor lesion where they robustly induce FOXP3 and other Treg signature molecules in human naïve CD4^+^ T cells which display enhanced FOXP3 stability and low expression of pro-inflammatory cytokines (208). Treg cells limit immune effector cell function via cytokines (209–212), direct lysis (213), inhibitory receptors on their cell surface (214–217), via metabolic disruption (218), by starving effector cells via depletion of local IL-2 (219) or indirectly by turning secondary cell types into suppressive ones i.e., IDO (220) and tolerogenic cytokine producing DCs with low costimulatory potential (221). Treg depletion dramatically reduced tumor growth or induced full remission (222–224). In contrast to conventional chemotherapeutic agents which also deplete T effector cells and may induce autoimmunity due to the systemic elimination of T-regs (225), CXCR4 targeting allows the specific targeting of Tregs, since intratumoral Tregs consistently express higher CXCR4 levels than CD4^+^CD25^−^ and CD8^+^ cells (226). In intraperitoneal papillary epithelial ovarian cancer, CXCR4 antagonism increased tumor cell apoptosis and necrosis, reduced intraperitoneal dissemination, and selectively reduced intratumoral FoxP3 Tregs (226). Superior immune responses as shown for CXCR4 antagonist BL-8040 is not solely owing to a selectively reduced recruitment of Treg cells into the tumor, and an increase in the number of immune and progenitor cells (227). CXCR4 antagonists have shown to also reverse Tregs' suppressive activity. Plerixafor and the antagonistic CXCR4 peptide R29 (228) inhibited Treg-suppressive activity significantly (by 10-fold) in Tregs from primary renal cancer specimens in which high numbers of activated Tregs (expressing CTLA-4/CXCR-4/PD-1/ICOS) were detected. A possible mechanistic explanation involves the demethylation of Treg-FOXP3 promoter (229). Thus, inhibition of Tregs by blocking SDF-1/CXCR4 is one of the major rationales for a better anti-tumor immune response via CXCR4 inhibition.

### Effector Cells

T effector (Teff) cells also constitutively express the chemokine receptor CXCR4. Besides T cell migration along SDF-1 gradients, CXCR4 after T cell receptor crosslinking is recruited to and accumulates at the immunological synapse, resulting in stronger T cell-APC interaction, shutdown of T cell responsiveness to chemotactic gradients, and in higher levels of T cell proliferation and IFN-γ production (230). *Vice versa*, the presence of competing external chemokine signals has been shown to disrupt the stability of T-APC conjugates as a result of impaired recruitment of the receptor to the immunologic synapse (231). CXCR4 confers the homing of antiviral T cell responses to bone marrow. Ablation of CXCR4 thus impairs memory cell maintenance due to defective homeostatic proliferation in the bone marrow niche, yet allows fully functional asymmetric cell fates after antigenic rechallenge in CD8^+^ T cells (232). Antitumoral activity was shown for CXCR4 antagonist BL-8040 in tumor bearing mice, where it induced robust mobilization of CD4^+^ and CD8^+^ T lymphocytes and DC in numbers that were significantly higher compared to tumor free naïve counterparts. The authors did not discriminate the lymphocytic population with respect of Teff/Treg ratio or CD8^+^ content though (233), yet showed in pre-clinical *in vivo* pancreatic cancer models, immune cells mobilized from the bone marrow into the circulation accumulate within the tumor lesion where they inhibit tumor growth. Such a dramatic effect on the intratumoral T cell compartment function is reflected in a study by Elda Righi (226) where CXCR4 antagonist AMD3100 by favorably modulating the intratumoral Teff/Treg ratio 6-fold, created a phenotype reminiscent of two studies that—although in different contexts—depleted intratumoral T-regs which highly significantly improved cytotoxic T-cell function in the tumor tissue and prolonged survival (234, 235). Along those lines, epigenetic down-regulation of SDF-1 expression in osteosarcoma has been demonstrated to impair cytotoxic T-cell homing to the tumor site (111). In contrast, SDF-1 overexpression by melanoma cells in the B16-ova melanoma model has been shown to chemo-repel antigen-specific cytotoxic T cells (236) suggesting a complex and fine-tuned control of Teff infiltration by SDF-1/CXCR4 signaling.

Chemokines control also the trafficking of developing and mature natural killer cells (NK) in the bone marrow (237). While several CCRs are expressed during progression from precursor to immature and mature NK cells CXCR4 was only detected on immature NK cells. Administration of the CXCR4 antagonist, AMD3100, induced strong reduction of mature NK and immature NK cells in the BM in a murine model and increased their number in blood and spleen, which suggests that this chemokine axis also regulates NK cell subsets localization in the bone marrow niche and their migration to the periphery for their maturation (238). Notably, genetic deletion of CXCR4 in myeloid cells in a melanoma mouse model fostered NK cell-mediated antitumor immunity suggesting indirect suppression of NK cell activity by CXCR4 signaling (239).

In summary, SDF-1/CXCR4 signaling affects most subsets of immune cells, the most prominent and clinically applied effect being the mobilization of HSCs by blocking CXCR4 as summarized in Table 2.

**Table 2:** (Patho)physiological role of SDF1/CXCR4 signaling and targeting in immune processes.

**Cell type**	**(Patho)physiological role of SDF-1/CXCR4 signaling**	**Effects of CXCR4 targeting**	**References**
Hematopoietic stem cells	Survival in BM		(178, 179)
	Decreased proliferation in BM		(180–182)
	Survival and growth of bone marrow stromal stem cells		(183)
		Mobilization	(184–187)
Dendritic cells	Dendritic cell development		(196, 197)
		Impaired immune response	(198)
Effector T cells	T cell proliferation and IFN-γ production		(230)
		Increased tumor infiltration	(233)
		Increased cytotoxicity	(234, 235)
Natural killer cells		Migration to periphery, maturation	(238)
		Increased NK cell mediated antitumor immunity	(239)
Regulatory T cells (Tregs)	Attraction to tumor lesions		(208)
		Reduced intratumoral Tregs	(226)
		Reduced Treg suppressive activity	(228)
Myeloid derived suppressor cells (MDSCs)	Induction and hampered immune response	Decreased metastases formation via reduced MDSCs	(203, 204)
	MDSC recruitment to tumors		(203, 204, 240)

*APC, antigen presenting cell; BM, bone marrow; MDSC, myeloid derived suppressor cells*.

### Combined Immunotherapy and CXCR4 Targeting

**Figure 2:**
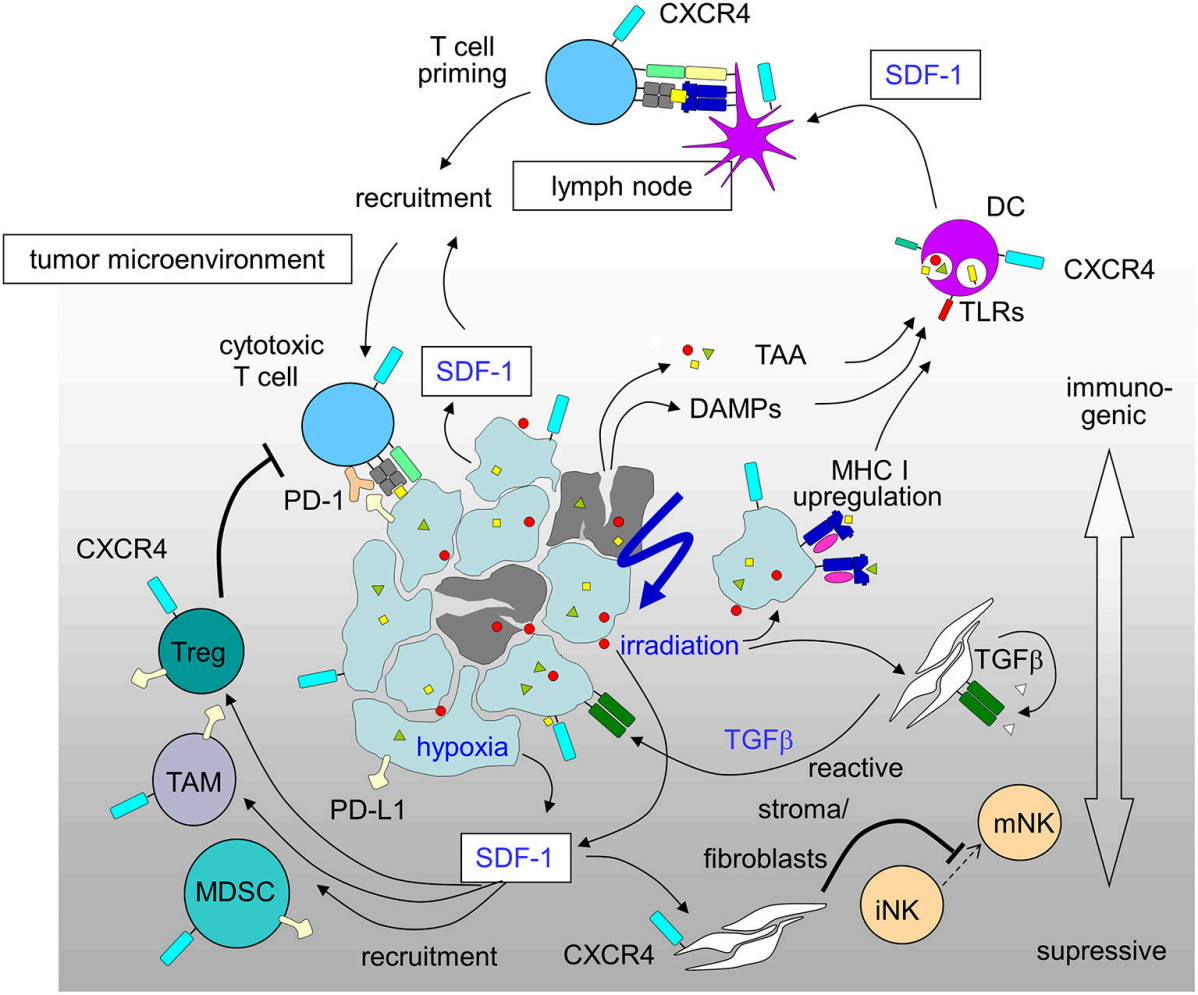
Immunosuppressive and immunostimulatory action of SDF-1/CXCR4 signaling in tumors induced by radiation-therapy and hypoxia (for details see text; DAMPs, danger-associated molecular patterns; DC, dendritic cells; MDSC, myeloid derived suppressor cell; PD-1, programmed cell death protein-1; PD-L1, PD-1 ligand; TAA, tumor-associated antigens; TAM, tumor-associated macrophage; Treg, regulatory T-cell).

The promotion of antitumor immunity by CXCR4-antagonists was reported for a mouse model of ovarian cancer (226) and in an orthotopic preclinical hepatocellular carcinoma (HCC) model where a CXCR4 antagonist was combined with a checkpoint inhibitor. In this HCC model multi-kinase-inhibitor sorafenib treatment-induced hypoxia fostered SDF-1 production, leading to the recruitment of immunosuppressive tumor-associated macrophages, myeloid-derived suppressive cells, and Tregs all with increased PD-L1 expression. CXCR4 antagonist plerixafor combined with anti-PD-1 therapy showed the most pronounced tumor growth delay, and was associated with increased intratumoral penetration and activation of CD8^+^ T lymphocytes (241). A novel strategy for the treatment of drug-resistant ovarian cancer combines chemotherapy to increase immunogenic cell death and virally delivered CXCR4 to reverse the immunosuppressive tumor microenvironment (242). Ovarian cancer of murine and human ovarian tumor variants resistant to paclitaxel and carboplatin were infected with oncolytic vaccinia virus expressing a CXCR4 antagonist and were +/– treated in combination with doxorubicin. The chemo-resistant variants' augmented expression of CXCR4 was associated with an increased susceptibility to viral infection and doxorubicin-mediated killing compared to parental counterparts *in vitro* and in tumor-challenged mice. Antitumor immune responses in this model culminated in the control of metastatic tumor growth and tumor-free survival. Mechanistically, the authors showed combination treatment increased apoptosis and phagocytosis of tumor material by DCs which efficiently induced adaptive antitumor immunity, reflected by increased intratumoral infiltration of antitumor CD8^+^ T cells and reduced immunosuppressive Tregs (242). Based on these results (Figure 2), the MORPHEUS clinical trials were started including treatment arms combining immune-checkpoint inhibitors with CXCR4 inhibition (NCT03193190, NCT03281369 and NCT03337698 for pancreatic cancer, gastric cancer and non-small cell lung cancer, respectively).

## Concluding Remarks

With combinatorial approaches of radiotherapy and immunotherapy on the rise, it is important to evaluate novel treatment strategies in radiation oncology with respect to tumor and radiation biology as well as immunologic effects. For SDF-1/CXCR4 targeting both perspectives provide a strong rationale for combination therapies. The SDF-1/CXCR4 axis plays pivotal roles in various aspects of tumor biology, and in particular in the stress response of tumors to ionizing radiation. In preclinical *in vivo* models CXCR4 targeting increases the efficacy of radiation therapy and blunts adverse effects, such as radiation-stimulated metastases and vasculogenesis. Mobilization of HSCs, a significant increase of immune and progenitor cells in the periphery that are able to migrate into the tumor and the selective targeting of Treg cells in the tumor lesion provide the rationale for an increased anti-tumor immune response upon CXCR4 inhibition. Preclinical mechanistic studies as well as translational and clinical evaluation of the role of the SDF-1/CXCR4 axis in the context of cancer radiotherapy and immunotherapy might lead to novel treatment strategies implementing SDF-1/CXCR4 targeting in this context using the small molecule inhibitors already approved for the use in patients and healthy donors for HSC mobilization.

## Author Contributions

FE and SH designed the concept and wrote the manuscript. LK wrote chapter SDF-1/CXCR4-Signaling and Radioresistance. MS wrote chapter SDF-1/CXCR4 Signaling as Druggable Target in Anti-cancer Therapy. LB wrote chapter Interference of Ionizing Radiation With Immune Responses. ES contributed to chapter SDF-1/CXCR4 Function in Tumor Biology. KS wrote chapter Physiologic Role of CXCR4 in the Immune System. DZ and all authors read and approved the manuscript.

### Conflict of Interest Statement

FE has a research collaboration with Merck KgAa. SH has a research collaboration with Novocure. DZ, FE have research and educational grants from Elekta, Philips, Siemens, Sennewald. The remaining authors declare that the research was conducted in the absence of any commercial or financial relationships that could be construed as a potential conflict of interest
